# Vision contributes to sex differences in spatial cognition and activity interests

**DOI:** 10.1038/s41598-022-22269-y

**Published:** 2022-10-21

**Authors:** Yiming Qian, Sheri A. Berenbaum, Rick O. Gilmore

**Affiliations:** Department of Psychology, The Pennsylvania State University, University Park, PA 16802 USA

**Keywords:** Psychology, Human behaviour

## Abstract

Sex differences in a variety of psychological characteristics are well-documented, with substantial research focused on factors that affect their magnitude and causes. Particular attention has focused on mental rotation, a measure of spatial cognition, and on activity interests. We studied whether sex differences in visual perception—luminance contrast thresholds and motion duration thresholds—contribute to sex differences in mental rotation and interest in male-typed activities. We confirmed sex differences in vision, mental rotation, and activity interests in a sample of 132 college students. In novel findings, we showed that vision correlated with mental rotation performance in women, that vision was a better predictor of individual differences in mental rotation than sex, and that contrast thresholds correlated with women’s interest in male-typed activities. These results suggest that sex differences in spatial cognition and activity interests may have their roots in basic perceptual processes.

## Introduction

Sex differences in a variety of psychological characteristics are well-documented, with substantial research focused on their causes and the factors that affect their magnitude^1–4^. Particular attention has focused on two broad domains: *spatial abilities* and *activity interests* and engagement, especially childhood toy play, adolescent and adult leisure activities, and occupational interests. Boys and men have better average spatial abilities than girls and women^5^. Both sexes are most likely to prefer and engage in gender-typed activities. Sex differences in spatial abilities and activity interests are larger for some tasks than for others^3,4^, but are generally moderate to large in magnitude^6,7^. Differences in spatial abilities and interests arise early in development^2,8,9^, have parallels in nonhuman animals (to toys in primates^10^; and to spatial abilities in several species^11,12^), and contribute to sex differences in occupational choice, particularly women’s underrepresentation in careers involving science, technology, engineering, and mathematics^13–16^ (STEM). Moreover, spatial abilities and activity interests correlate^17,18^.

A key question concerns the proximal mechanisms that drive sex differences in spatial abilities and activity interests. Previous work has focused on biological (especially hormonal) and social causes. Little work has focused on the perceptual factors that might mediate those causes. As with spatial abilities and interests, sex differences in vision are influenced by both biology and experience^19,20^. Several lines of evidence suggest that males and females differ in multiple aspects of visual processing^21,22^. Compared to women, men show enhanced contrast sensitivity particularly at higher spatial frequencies^23,24^. Sex differences in contrast sensitivity emerge early in life: Male advantages in contrast sensitivity were found at two-months of age^25^ and in 4–12 year-old children^26^. Motion processing also shows male advantages across a range of displays and tasks^21,27,28^, and these also emerge early in life. Male preferences for propulsive movement were found in 6-to 9-month infants^29^, and a male advantage in detecting visual motion has been observed in preschool and school-age children^30,31^.

Could sex differences in vision help explain sex differences in spatial ability or activity interests? Shaqiri et al.^21^ suggested that differences in visual perception could account for a number of sex differences observed in visually-based cognitive tasks. Mental rotation is a form of spatial ability that is a promising candidate for testing this claim: It is a visually dependent task; it shows the largest sex difference among spatial tasks^5^; the magnitude of this difference increases over development^32^; performance can be altered by experience^5,33^; and it associated with other abilities both in the lab (e.g., prospective memory^34^) and in the real world (e.g., STEM^5^). Similarly, activities can vary in their perceptual demands, so sex differences in visual abilities might relate to differential participation or interest in these activities.

Here, we studied how two measures of visual perception that have shown sex differences are related to mental rotation and activity interests. We had two goals. First, we sought to replicate previously reported sex differences in vision^23,28^, hypothesizing that men would have lower contrast thresholds and lower motion duration thresholds than women. We also expected to confirm well-established sex differences in mental rotation and activity interests, with men scoring higher than women on mental rotation, and preferring male-typed activities, and women preferring female-typed activities. Second, we examined correlations between vision and mental rotation and activity interests, hypothesizing that, within-sex, contrast thresholds and motion duration thresholds would be negatively correlated with mental rotation and with male-typed activity interests, and that sex differences in vision would account for sex differences in mental rotation and activity interests. We further predicted that contrast thresholds and motion duration thresholds would be positively correlated with each other.

## Methods

### Participants

We collected data from 132 undergraduates (30 males) who participated for credit in psychology courses. The sex bias reflected the demographics of the subject pool and course enrollment. The vast majority of participants were 18–19 years old, first-year college students, and right-handed; they varied in race and ethnicity. All participants had visual acuity better than 20/40 as measured by the Snellen visual acuity test and had no deficits in stereo vision or color vision. Informed consent to participate was obtained from all participants under the procedures approved by the Pennsylvania State University Internal Review Board (STUDY00013345; Study title: “Individual differences in visual perception in adults”). All methods were performed in accordance with relevant guidelines and regulations. Data were missing for two women on the contrast task, four women on the motion duration task, and one woman on measures of mental rotation and activity interests.

### Measures

We measured contrast thresholds and temporal duration thresholds separately using a PC running Windows 10 and using custom software written in Python using the Psychopy 3.2.4 framework. All code used to generate the displays is hosted on GitHub (https://github.com/gilmore-lab/sex-differences-project/). For both measures, sinusoidal grayscale gratings were presented within a 3.5 deg diameter circular aperture in the center of a mid-gray background with a mean luminance of 20.15 cd/m^2^. Displays were presented on a Mitsubushi monitor (Diamond Pro 2060u) with a screen resolution of 800 × 600 pixels and a screen refresh rate of 85 Hz. Participants were seated at a fixed viewing distance of 60 cm, making the full display 36.6 deg (horizontal) by 27.9 deg (vertical).

A brief instruction period and ten practice trials were followed by four runs of 30 trials each. On each trial of each run, contrast or duration was adjusted according to the participant’s performance on the previous several trials following a three-down and one-up staircase procedure using the QUEST algorithm. The guessing rate and lapse rate were set at 0.5 and 0.01, respectively. The total time to complete both psychophysical tasks was about 8 min. The specific parameters were chosen to maximize sex differences observed previously^23,28^ and to equate parameters across the contrast and motion tasks as much as possible.

#### Contrast threshold

Replicating the published procedure of Abramov et al.^23^, for each trial, a central fixation cross appeared for 0.45 s followed by a blank screen for 0.4 s, and then a single horizontal or vertical 12 cyc/deg grating appeared for a 2 s response period. In each trial, the sinusoidal grating was defined by a full-width at half-maximum (FWHM) temporal envelope (contrast increased linearly from zero for 0.5 s, remained at full contrast for 1 s, and decreased to zero for 0.5 s). Overall grating contrast varied at a temporal frequency of 4 Hz, creating a flickering appearance. Participants were instructed to report the grating’s orientation (horizontal/vertical) using the corresponding arrow keys on the keyboard using their dominant hand and were given unlimited time to respond. Auditory feedback was provided with each correct response. The interstimulus interval was 0.15 s.

#### Motion duration threshold

We measured a motion duration threshold—the minimum temporal duration needed to correctly detect the direction of motion. Replicating the published procedures of Murray et al.^28^, for each trial, a central fixation appeared for 0.85 s and participants pressed a button when they were ready to see the next grating stimulus; a short 0.15 s blank screen appeared, followed by a 1.2 cyc/deg vertical grating at a Michelson contrast level of 98%. The grating drifted leftward or rightward at a 4 Hz temporal frequency (3.33 deg/s). The minimum temporal duration for a single video frame was 11.8 ms (1/85 Hz). Since the generation of motion requires at least two video frames, the minimum display interval was 23.5 ms. The gratings appeared for a variable duration, defined by a FWHM temporal envelope controlled by the QUEST staircase algorithm. Once the grating displays appeared and disappeared, participants had unlimited time to indicate the perceived leftward or rightward direction of motion by pressing the corresponding arrow keys on the keyboard. Auditory feedback was provided with each correct response in each trial. The interstimulus interval was chosen randomly from a uniform distribution between 0.25 and 0.5 s.

#### Mental rotation

We used a computer-based Mental Rotation Test developed by Vandenberg and Kuse^35^, adapted from Shepard and Metzler’s task^36^. Each of 20 items contained pictorial two-dimensional images of a three-dimensional target figure and four other three-dimensional figures. The task was to identify the two figures that were rotations of the target figure in three-dimensional space, with a time limit of 10 min. The participant’s score was the number of correct items out of 40 possible.

#### Vocabulary

We measured vocabulary, an aspect of verbal ability that correlates well with overall intelligence but does not show sex differences^37^. We used the Advanced Vocabulary Test^38^, with two parts of 18 items each. For each item, participants were given a target word and asked to choose the word with the same or nearly the same meaning from a list of five choices. Scores were corrected for guessing, by calculating the number of correct answers minus a fraction (0.25) of the number of incorrect answers.

#### Activity interests

Participants rated their degree of preference for each of 60 leisure activities (for example, “Home electronics”, “Cooking”, “Clothes shopping”) on a 5-point scale from strongly dislike (1) to strongly like (5)^39^. Prior work with this measure^39^ has shown that some of these activities are strongly sex-typed: There were 15 male-typed activity items (*alpha* = 0.79) and 22 female-typed activity items (*alpha* = 0.80).

### Procedure

Testing was conducted in the laboratory in a single session lasting 30–45 min. After informed consent was given, participants performed a visual screening test, followed by two psychophysical measures of visual perception (in random order across participants). Participants then completed computerized measures of mental rotation and vocabulary, answered questions about activity interests, major field of study, year in school, age group, race, handedness, and use of glasses or contacts.

### Data analyses

Prior to the start of data collection, we conducted a power analysis and shortly after data collection began filed a preregistered analysis plan (https://aspredicted.org/5iv9a.pdf). Based on prior research with our participant pool, our plan envisioned a sample of *n* = 300 with unequal numbers of men (*n* = 45) and women (*n* = 255). Based on those assumptions and a Bonferroni-corrected alpha of 0.0125, we had 0.8 power to detect minimum effect sizes of *d* ≥ 0.50 and |*r|*≥ 0.20. Restrictions on in-person data collection due to Covid-19 forced premature study closure and resulted in a smaller sample than planned. The validity of post hoc power analyses has been questioned^40^, but our pre-data collection analysis evaluated a range of sample sizes, including *n* = 150, and a range of male/female participant ratios. That analysis showed that we had 0.8 power to detect *d*’s of 0.55–0.6.

For each participant’s run, we estimated a contrast or duration threshold that predicted 80% correct performance by fitting a Weibull function using maximum likelihood estimation. Single runs with less than 68% accuracy (the upper limit of a 95% confidence interval for a 50% guess rate) were excluded. We then calculated a median threshold across all the remaining runs for each task and each participant. Finally, we log-transformed thresholds to make the distributions more symmetric and thus suitable for parametric analyses. We note that lower perceptual thresholds mean higher sensitivity.

To confirm predicted sex differences in vision, mental rotation, and male-typed activity interests, we used one-tailed *t*-tests; for vocabulary, we used a two-tailed *t*-test because we did not predict a sex difference. To examine links between the vision measures and mental rotation, we examined correlations within sex using complete observations on those variables (pairwise deletion). In line with calls to focus on effect sizes rather than null-hypothesis significance testing^41^, we report effect sizes with confidence intervals. We estimated mean sex differences (male minus female) adjusted for unequal sample sizes (*d*_*adj*_) using a statistic sometimes called Hedges *g*, described associations with correlation coefficients, and computed confidence intervals using the *esvis*^42^ and *esci*^43^ packages. Statistical summaries follow Lakens’ recommendations^41^.

To explore the relative influence of contrast thresholds, motion duration thresholds, and sex on mental rotation scores, we conducted a sequential regression model-fitting procedure to determine which model fit the data best and with the fewest parameters. Starting with a full model including all interactions, we eliminated predictors whose values did not meet a *p* < 0.1 criterion in a test of whether the estimated predictor weight exceeded zero, and we compared models using ANOVA. If the simpler model with fewer parameters had the same or better fit than the more complex model, we selected the simpler model. We then conducted a relative weight analysis^44^ to estimate the relative contribution of the predictors that remained in the best-fitting model to the total variance explained by that model.

R Markdown documents that describe all data cleaning, analysis, and visualization procedures can be found on GitHub (https://github.com/gilmore-lab/sex-differences-project/).

### Conference presentation

Portions of these findings were presented at the 2021 Vision Sciences Society meeting.

## Results

**Figure 1:**
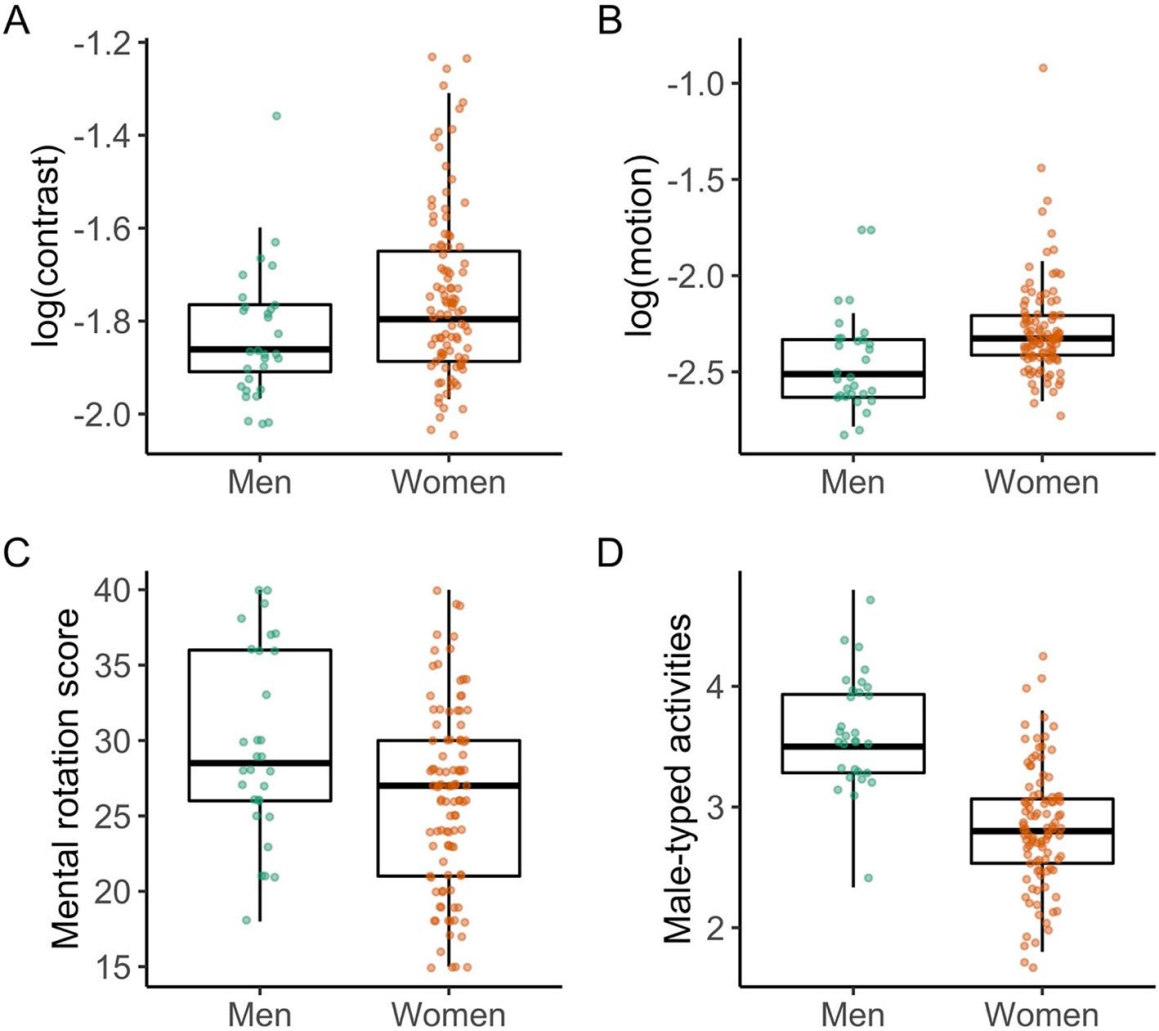
Sex differences in vision, mental rotation, and interest in male-typed activities. Jittered data from individual participants for (**A**) log contrast thresholds, (**B**) log motion duration thresholds, (**C**) mental rotation scores (total correct out of 40 possible), and (**D**) average interest in 15 male-typed activities. Larger thresholds reflect poorer performance. An *alpha* value of 0.5 was applied during plotting to compensate for overlapping points.

All predicted sex differences were confirmed (Fig. 1). Compared to men (M =  − 1.83, SD = 0.18), women (M =  − 1.74, SD = 0.13) showed higher log contrast thresholds (Fig. 1A), *t*(128) =  − 2.49, *p* = 0.007, *d*_adj_ =  − 0.51, 95% CI [− 0.94, − 0.11], higher log motion duration thresholds (Fig. 1B) (women: M = − 2.27, SD = 0.25; men: M =  − 2.45, SD = 0.26), *t*(126) =  − 3.51, *p* < 0.001, *d*_adj_ =  − 0.73, 95% CI [− 1.16, − 0.32], lower mental rotation scores (Fig. 1C) (women: M = 26.21, SD = 6.00; men: M = 29.67, SD = 6.22), *t*(129) = 2.75, *p* = 0.003, *d*_adj_ = 0.57, 95% CI [0.16, 1.00], and lower interest in male-typed activities (Fig. 1D) (women: M = 2.84, SD = 0.50; men: M = 3.62, SD = 0.49), *t*(129) = 7.58, *p* < 0.001, *d*_adj_ = 1.57, 95% CI [1.14, 2.05]. Also as expected, we found no meaningful difference in vocabulary (Fig. S1), (women: M = 9.17, SD = 4.64; men M = 9.92, SD = 5.42), *t*(129) = 0.75, *p* = 0.456, *d*_adj_ = 0.15, 95% CI [− 0.25, 0.57].

**Figure 2:**
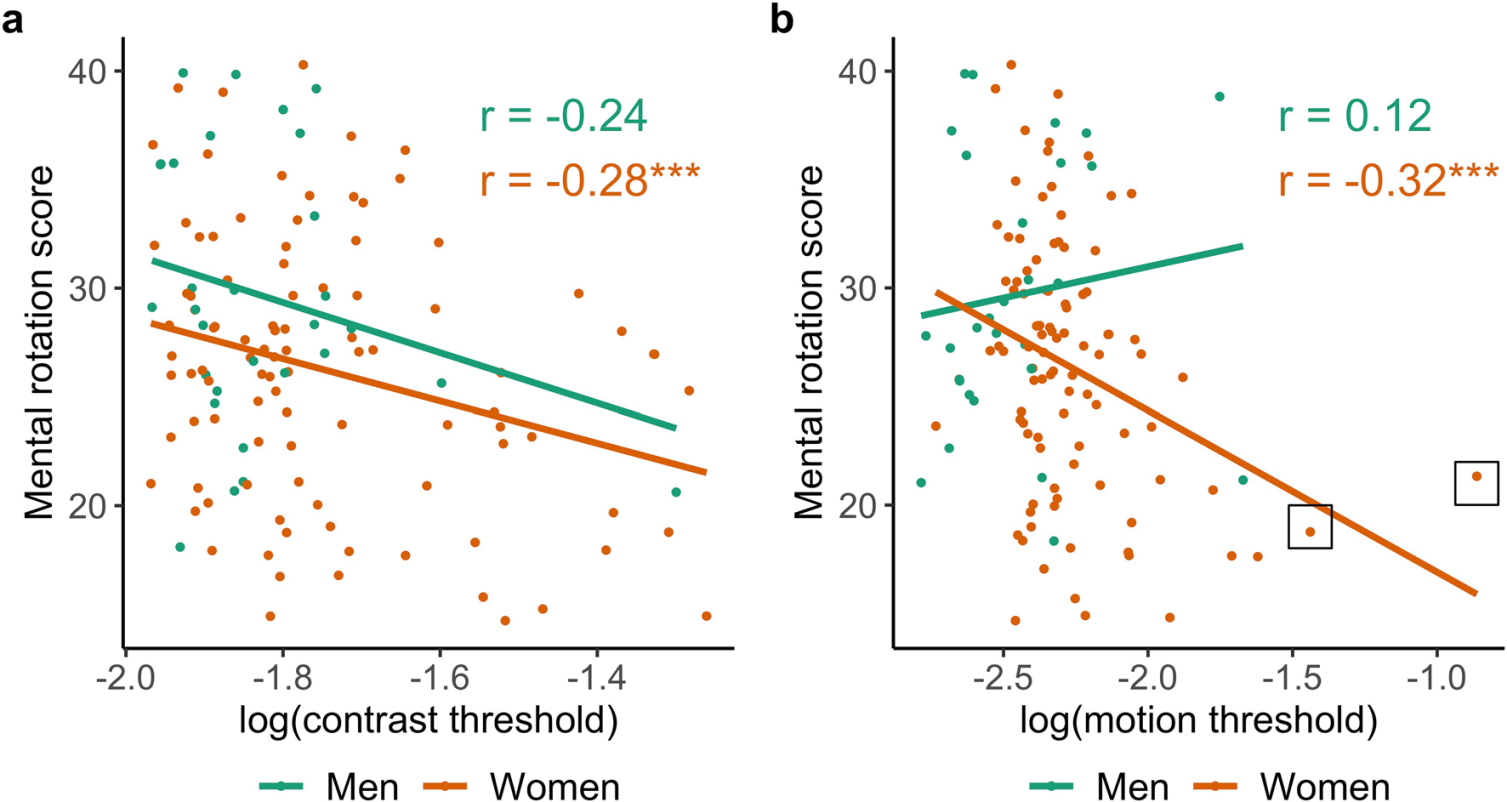
Associations between mental rotation scores and vision measures. Each point represents an individual participant’s mental rotation scores as a function of (**A**) log contrast thresholds and (**B**) log motion duration thresholds. Two female participants whose log motion duration thresholds exceeded 3 SD from the combined sample mean are indicated with squares. Larger thresholds reflect poorer performance. The figure shows that vision thresholds correlate with mental rotation scores, especially in women. Correlation significantly less than 0, ***p < 0.005.

**Figure 3:**
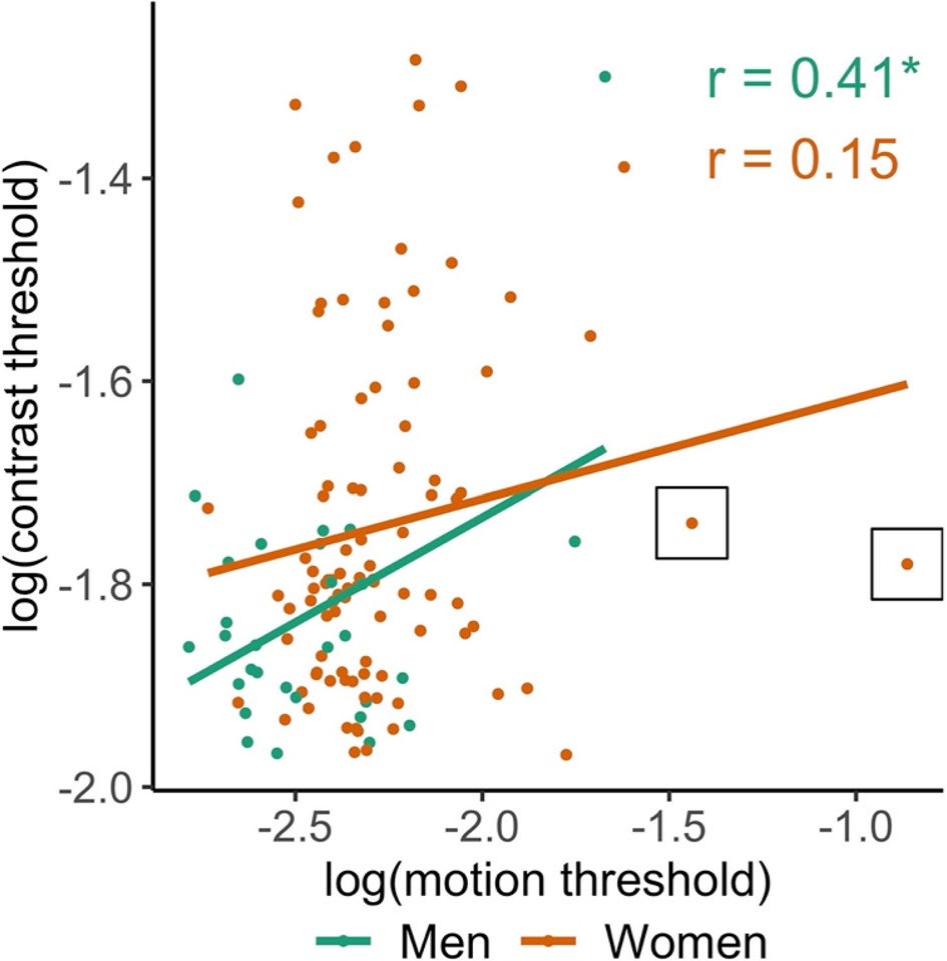
Association between log contrast thresholds and log motion duration thresholds. Each point represents an individual participant’s log contrast thresholds as a function of their log motion duration thresholds. Two female participants whose log motion duration thresholds exceeded 3 SD from the combined sample mean are indicated with squares. Larger thresholds reflect poorer performance. The figure shows that vision thresholds correlate with one another. Correlation significantly greater than 0, *p < 0.05.

The predicted negative correlation between log contrast threshold and mental rotation was found for women (Fig. 2A), *r*(97) =  − 0.28, *p* = 0.002, 95% CI [− 0.46, − 0.09]. The correlation in men *r*(28) =  − 0.24, *p* = 0.096, 95% CI [− 0.56, 0.13] was also negative and of similar magnitude, but the 95% confidence interval included zero (Fig. 2A). The predicted negative correlation between log motion duration thresholds and mental rotation was found for women (Fig. 2B), *r*(95) =  − 0.32, *p* < 0.001, 95% CI [− 0.49, − 0.13] but not men *r*(28) = 0.12, *p* = 0.737, 95% CI [− 0.25, 0.46] (Fig. 2B). A predicted negative correlation between log contrast thresholds and interest in male-typed activities was found for women (Fig. S2), *r*(97) =  − 0.19, *p* = 0.027, 95% CI [− 0.38, 0.00] but not men *r*(28) = 0.21, *p* = 0.867, 95% CI [− 0.16, 0.53]. The predicted negative correlation between log motion thresholds and interest in male-typed activities was not found in women, *r*(95) = 0.03, *p* = 0.629, 95% CI [− 0.17, 0.23] or men, *r*(28) = 0.07, *p* = 0.653, 95% CI [− 0.29, 0.42]. Log contrast and log motion duration thresholds were positively correlated in men *r*(28) = 0.41, *p* = 0.013, 95% CI [0.05, 0.67] and in women *r*(94) = 0.15, *p* = 0.078, 95% CI [− 0.06, 0.34], but, for women, the 95% confidence interval included zero (Fig. 3).

To explore whether the vision measures accounted for sex differences in mental rotation performance, we used linear regression and a sequential model comparison procedure (shown in Table 1). The best-fitting model (Model 1) included both vision measures, sex, and a sex by motion interaction term. Specifically, Model 1 fit better than a model without the sex and sex by log motion interaction terms, *F*(2, 122) = 4.01, *p* = 0.021 (Model 2), a model without log motion threshold, *F*(2, 122) = 4.66, *p* = 0.011 (Model 3), and a model with sex alone, *F*(3, 123) = 5.77, *p* = 0.001 (Model 4).

**Table 1:** Comparisons among regression models predicting mental rotation scores.

Models and predictors	Est (SE)	*F*	${\eta }_{p}^{2}$	*R*^2^	*R*^2^_adj_
**Model 1**				17.25	14.49
Log contrast threshold	− 8.30 (3.21)	6.67*	0.05		
Log motion threshold	− 6.56 (2.33)	3.65^+^	0.03		
Sex (male)	28.82 (11.36)	2.36	0.02		
Log motion threshold $\times$ sex	11.17 (4.69)	5.67*	0.05		
**Model 2**				11.72	10.27
Log contrast threshold	− 8.47 (3.24)	6.82*	0.05		
Log motion threshold	− 4.73 (2.03)	5.44*	0.04		
**Model 3**				10.83	9.37
Log contrast threshold	− 8.89 (3.24)	7.55**	0.06		
Sex (male)	2.56 (1.25)	4.17*	0.03		
**Model 4**				5.31	4.54
Sex (male)	3.30 (1.26)	6.90**	0.05		

Parameter estimate summaries for a model predicting mental rotation from sex, log contrast thresholds, log motion thresholds, and a sex by log motion threshold interaction (Model 1), a model including vision measures alone (Model 2), a model including log contrast thresholds and sex (Model 3), and a model including sex alone (Model 4). ^+^*p* < 0.1, *p < 0.05, **p < 0.01.

A subsequent relative weight analysis showed that Model 1 accounted for 17% of the total variance in mental rotation: log contrast thresholds accounted for 6%, 95% CI [1%, 14%]; log motion thresholds accounted for 4%, 95% CI [0.5%, 12%]; the sex by log motion threshold interaction term accounted for 4%, 95% CI [0.2%, 14%], and sex accounted for 3%, 95% CI [0.2%, 11%]. Alternatively, log contrast thresholds accounted for 35% of the *explained* variance, log motion thresholds 25%, the sex by log motion threshold interaction 21%, and sex 19%. Causal mediation analyses (Supplemental Material) confirmed that log motion and log contrast mediated the relationship between sex and mental rotation.

Regression analyses can be sensitive to outliers, so we explored the influence of extreme scores by trimming cases where log contrast thresholds, log motion duration thresholds, or mental rotation scores exceeded three standard deviations from the total sample mean. Two female participants had motion duration thresholds that exceeded this threshold. Removing these participants left the results unchanged, with one exception: The 95% confidence interval for the correlation between log contrast and log motion duration thresholds in women now excluded zero, *r*(92) = 0.21, *p* = 0.020, 95% CI [0.01, 0.40].

## Discussion

We provided novel evidence that sex differences in visual perception contribute to sex differences in mental rotation and to interest in male-typed activities. We replicated findings that men have better vision—lower contrast thresholds and shorter motion duration thresholds—than women, with moderate effect sizes; we also confirmed expected mental rotation with moderate effect sizes and sex differences in male-typed activity interests with very large effect sizes. The observed magnitudes of sex differences in contrast thresholds^23^, motion duration thresholds^28^, mental rotation^32^, and activity interests^7^ were comparable to those observed in prior studies.

Mental rotation scores were correlated with vision—higher mental rotation scores with lower contrast thresholds (in women and men) and shorter motion duration thresholds (in women)—with moderate effect sizes. These results were unchanged after excluding women with extreme motion duration thresholds. Further, combined scores on the two vision tasks led to better predictions of individual differences in mental rotation performance than did sex alone, and vision measures accounted for more of the variance in mental rotation scores than did sex. We also predicted and found a negative correlation between interest in male-typed activities and contrast thresholds in women, but did not find this relationship with motion thresholds. Predicted associations were weaker in men than in women, most likely due to low statistical power. Contrast and motion duration thresholds were positively correlated in men, and also in women when two extreme scores were excluded, with small to medium effect sizes.

These results need to be considered in light of some methodological limitations. First, Covid restrictions led us to test about half the sample originally planned. This reduced our statistical power, so it is noteworthy that the key hypothesized effects were detected. The sample of men is smaller than planned, but large enough to enable the detection of sex differences. Nevertheless, it is important to be cautious in interpreting null effects, especially in men. Second, some participants were missing data, so our results are based on somewhat different subsets of the full dataset; however, the amount of missing data was small and unlikely to bias results. Third, the measure of activity interests is limited in several ways: It is heterogeneous and somewhat dated; self-reported interests are an imprecise measure of actual participation; and scale items were not specifically chosen to map onto the visual skills assessed. Correlations between mental rotation and male-typed activity interests were low and confidence intervals included zero. These limitations may account for inconsistent links found between vision and activity interests. Fourth, sex differences in contrast sensitivity appear to depend on display parameters: Previous studies that failed to find male advantages in contrast sensitivity^21,24^ used lower spatial frequencies than we did. In contrast, a wide range of motion processing tasks and display parameters show sex differences favoring males.

Our findings raise several questions worth further study. One concerns the ways that these particular visual abilities influence mental rotation or activity interests. The measures of vision used in the study were based on previous reports of sex differences, without specific consideration of how they might relate to the core processing components of mental rotation or to activity interests. Nevertheless, we conjecture that enhanced abilities to detect low contrast/high spatial frequency information or motion direction at short durations might facilitate the ability to perceive the 3D orientation of static objects and imagine them moving in three dimensions—a common strategy in mental rotation tasks.

Two sets of evidence support these conjectures. First, neuroimaging results show that cortical areas linked to vision for detail (e.g., high spatial frequencies) and visual motion processing are activated in mental rotation tasks^45^. So, mental rotation may depend on neural circuits that are engaged in lower level perceptual processes that in turn differ by sex. Second, high achieving athletes who play interceptive sports like basketball have higher visual acuity, contrast sensitivity, and shorter reaction times than athletes who play other sports^46^. These effects are more pronounced in older, more experienced, and more skilled athletes. Action video game play enhances contrast sensitivity^20^ and some types of motion discrimination^47^. Playing Tetris enhances mental rotation^48^. Thus, sex-differentiated visual biases might maintain or widen already-existing biological predispositions to sex-typed activity interests, which could in turn foster spatial ability^49^, or serve as a basis for self-socialization–with reciprocal associations between visual perception and other characteristics.

Another question concerns the ways in which vision also influences other aspects of spatial cognition that show sex differences^3^ and potential differential effects. For example, mental rotation has been linked to memory capacity^34,50^ and to success in STEM careers^5,16^, but small scale (e.g., mental rotation) and large-scale (e.g., navigation) spatial skills are distinct^51^.

Our findings suggest that attention to and careful measurement of basic visual processing will help to characterize individual differences in important cognitive skills and understand their emergence. We recommend that future work explore the links between various spatial tasks and the variety of vision tasks that show sex differences^21,46^. Spatial abilities in general are important for psychological development, and understanding how they are influenced by visual perceptual processes may contribute to enhancing them and increasing the participation of girls and women in STEM careers^3,5,13,15,32–34^.

In conclusion, evidence from this study shows that sex differences in vision play a previously undiscovered role in sex differences in other psychological domains. We found that sex differences in contrast thresholds and motion duration thresholds contribute to sex differences in mental rotation, and perhaps also to activities that are typically more popular with men than with women. The results highlight the role that basic perceptual processes might play in a broad range of behaviors and the importance of considering them in studies of individual and group differences and their development.

## Supplementary Information

Supplementary Information.

## Data Availability

The study reported in this article was preregistered (https://aspredicted.org/5iv9a.pdf), but additional analyses were performed that were not formally preregistered. All processed data and analysis code have been made available via GitHub and complete analysis scripts can be accessed at https://github.com/gilmore-lab/sex-differences-project. The questionnaire materials used in these studies are widely available, and code used to generate psychophysical experiments are available on https://github.com/gilmore-lab/sex-differences-project/.
