# Supplementary material for: Vision contributes to sex differences in spatial cognition and activity interests
Source: Sci Rep. 2022 Oct 21;12:17623. doi: 10.1038/s41598-022-22269-y (PMC9586946; doi:10.1038/s41598-022-22269-y)
Supplement: Supplementary file 1 — Supplementary Information. [file 41598_2022_22269_MOESM1_ESM.docx]

# Supplemental Material

## Mediation Analysis

We conducted a mediation analysis to estimate the causal influence of sex and the two vision measures on mental rotation scores. Using *z*-score transformed scores for mental rotation, log contrast thresholds, and log motion thresholds, we tested two models. One model tested the direct effect of sex on mental rotation and the indirect effect of sex on mental rotation via log contrast thresholds. The other model tested the indirect effect of sex on mental rotation via log motion thresholds. In both cases, we tested the significance of the indirect effect via bootstrapping with the *mediation* package in R. Unstandardized indirect effects were computed for each of 1,000 bootstrapped samples, and the 95% confidence interval was computed by determining the indirect effects at the 2.5^th^ and 97.5^th^ percentiles.

The indirect effect of sex on mental rotation via log contrast thresholds was (.50)*(-.24)=-.12. The bootstrapped indirect effect was -.12, 95% CI = [-.24, -.02], *p* = 0.012. Similarly, the indirect effect of sex on mental rotation via log motion thresholds was (.18)*(-.79)=-.14. The bootstrapped indirect effect was -.14, 95% CI = [-.33, -.01], *p* = 0.03. Thus, we conclude that vision measures mediate the relationship between sex and mental rotation performance in this sample.

## Figure S1

*Vocabulary Scores by Sex*


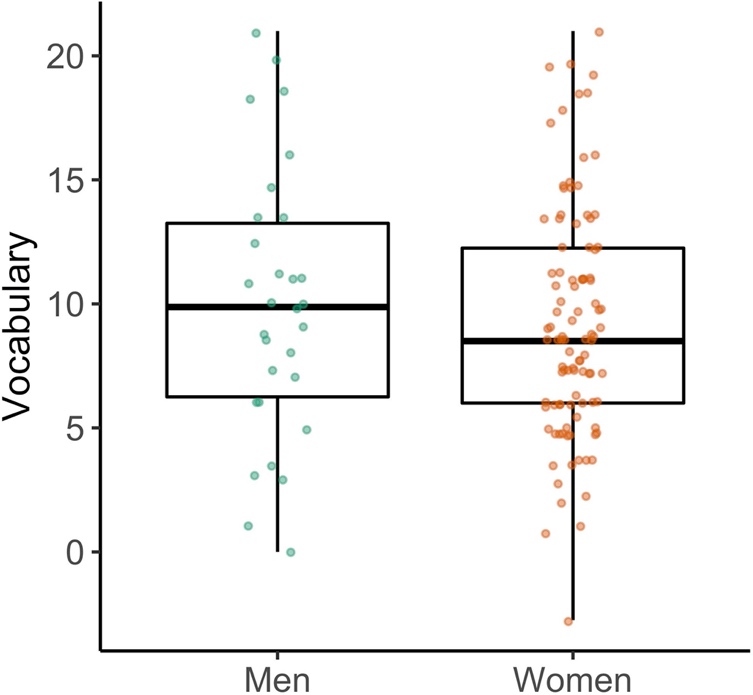


*Note*: Each point represents an individual participant’s vocabulary score. The data were jittered and an *alpha* value of 0.5 was applied to compensate for overlapping points.

## Figure S2

*Association Between Interest in Male-typed Activities and Contrast Thresholds*


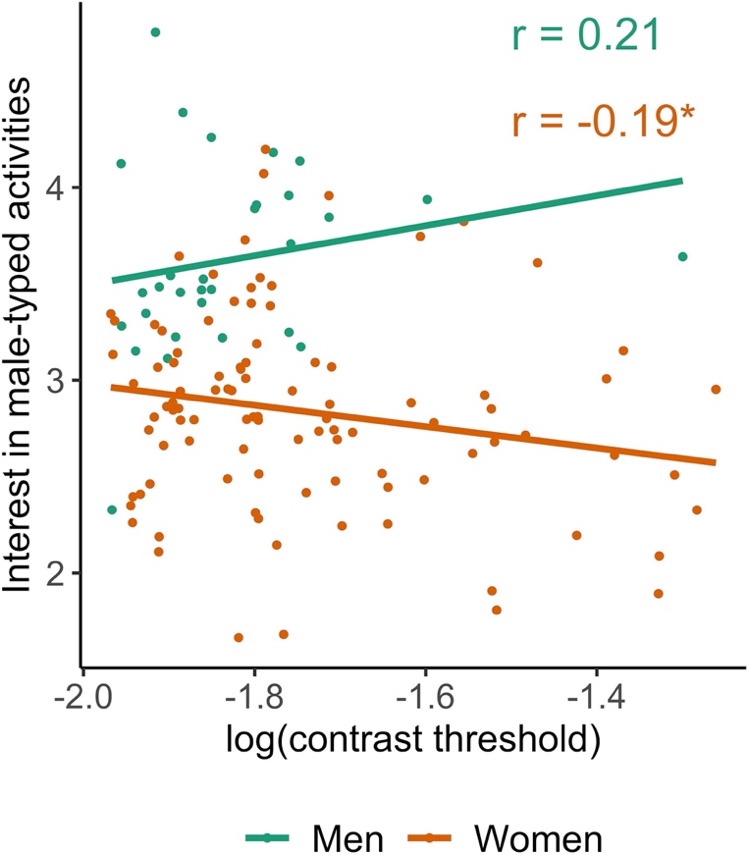


*Note*: Each point represents an individual participant’s average interest in male-typed activities as a function of their log contrast thresholds. Larger contrast thresholds reflect poorer performance. The figure shows that contrast thresholds correlate with interest in male-typed activities in women. Points were jittered before plotting to reduce overlap. Correlation significantly less than 0, ** p* < .05.
